# Brazilian guide for the diagnosis of severe community-acquired pneumonia and hospital-acquired pneumonia

**DOI:** 10.1016/j.clinsp.2025.100793

**Published:** 2025-09-29

**Authors:** Jorge Luiz Mello Sampaio, Alexandre Cunha, Daniel Wagner de Castro Lima Santos, Alvaro Pulchinelli Junior

**Affiliations:** a Faculdade de Ciências Farmacêuticas da Universidade de São Paulo, São Paulo, SP, Brazil; b Sabin Diagnóstico e Saúde, São Paulo, SP, Brazil; c Hospital Sírio Libanês, São Paulo, SP, Brazil; d Instituto D'Or de Pesquisa e Ensino (IDOR), Rio de Janeiro, RJ, Brazil; e Hospital Universitário da Universidade Federal do Maranhão (HU-UFMA), Ebserh, São Luis, MA, Brazil; f Sociedade Brasileira de Patologia Clínica/Medicina Laboratorial (SBPC/ML), Rio de Janeiro, RJ, , Brasil; g Clínica Médica e Medicina Laboratorial, Universidade Federal de São Paulo (UNIFESP), São Paulo, SP, Brazil; h Laboratório Fleury, São Paulo, SP, Brazil

**Keywords:** Pneumonia, Molecular panels, Diagnosis, Guidelines

## Abstract

Pneumonia is one of the main causes of intensive care unit admission and death in Brazil. There is a need to standardize the use of microbiological tests for the diagnosis of pneumonia.

**Objective:**

To evaluate microbiological diagnostic data for severe pneumonia. Method: Comparative studies that evaluated the microbiological diagnosis of community pneumonia and hospital-acquired pneumonia were analyzed. The literature review was guided by two questions and used flowcharts prepared by experts.

**Results and Discussion:**

Diagnostic tests for pneumonia should be requested based on the severity of the disease, the patient´s immune status, and the risk of infection by multidrug-resistant bacteria. Gram staining may aid in excluding *S. aureus* pneumonia and evaluating the quality of respiratory samples, while culture of these clinical samples may identify the infectious agent in up to half of the cases and allow antimicrobial susceptibility testing to be carried out. Blood cultures have a low sensitivity but may be useful for diagnosing extrapulmonary infections or situations involving sepsis or septic shock. Rapid molecular panels have high sensitivity and specificity for viral and bacterial targets for both types of pneumonia, and their use can reduce the length of hospital and intensive care stays and allow optimization of antimicrobial therapy. RT-PCR is highly accurate in diagnosing SARS-CoV-2 pneumonia.

**Conclusion:**

The rational use of molecular panels for the diagnosis of severe pneumonia can reduce the length of hospital and intensive care stays and 90-day mortality.

## Highlights

• No consensus on the use of molecular panels in the diagnosis of severe pneumonia.
• Algorithm for the use of laboratory tests for the diagnosis of pneumonia.
• Molecular panels in the diagnosis of pneumonia have reduced hospitalization time.
• Molecular panels in pneumonia reduced the length of stay in intensive care units.

## Introduction

Admission rates to Intensive Care Units (ICUs) have increased with higher life expectancy, access to new therapies for chronic diseases, and advances in health care^1^ Additionally, there has been a significant increase in antimicrobial resistance rates among *Enterobacterales, Pseudomonas* and *Acinetobacter,* and the overuse of large-spectrum antimicrobial agents. In recent years, companies have launched new rapid multiplex molecular tests for the diagnosis of infectious diseases and the detection of key antimicrobial resistance genes. If correctly used, they could be an excellent tool for improving diagnosis and consequently patient care. This scenario represents a considerable challenge for health care systems, especially in countries with limited resources.

Lower respiratory tract infections are the fourth leading cause of death worldwide. Before the COVID-19 pandemic, the World Health Organization estimated that the yearly number of deaths caused by lower respiratory tract infections worldwide was approximately 2.5 million. Notably, lower respiratory tract infections are the most frequent cause of death among infectious diseases^2^

Pneumonia is the most common infectious disease of the lower respiratory tract. Community-Acquired Pneumonia (CAP) and Hospital-Acquired Pneumonia (HAP) have different characteristics and impacts and high rates of morbidity, mortality, and significant economic costs^3^, ^4^, ^5^ CAP is defined as an infection of the pulmonary parenchyma that is acquired and occurs outside of hospital settings. It can affect individuals of all age groups, but is more frequent for those aged 55 or over. Viral or bacterial agents cause most of these infections^3^^,^^5^ Among viral agents, influenza, parainfluenza, rhinovirus, adenovirus, metapneumovirus, and coronavirus are the most frequent agents in adults, while respiratory syncytial virus is the most frequent agent in young children. *Streptococcus pneumoniae* is the most common bacterial pathogen, followed by *Staphylococcus aureus* and *Mycoplasmoides pneumoniae*^6^ Some clinical conditions may increase the risk of pneumonia, including prolonged use of steroids, bone marrow or solid organ transplantation, chemotherapy, pregnancy, AIDS, asthma, bronchiectasis, chronic obstructive pulmonary disease, uncompensated diabetes mellitus, heart failure, and central nervous system disorders such as dementia and Parkinson´s disease. The clinical diagnosis of CAP is based on the presence of symptoms and signs such as cough, sputum, shortness of breath and chest pain, together with systemic manifestations such as confusion, headache, sweating, chills, myalgia, and fever above 37.8 °C^3^ The severity of CAP is an important factor to be evaluated to determine if the patient will be treated as an outpatient or inpatient, the diagnostic procedures and tests that should be requested by the attending physician, and the choice of the best antimicrobial therapy, if indicated. Social and economic factors must also be considered when deciding to treat patients as outpatients or inpatients^3^ Additionally, CAP is one of the main causes of admission to ICUs, especially among elderly people, and is one of the main causes of death in hospitalized patients in Brazil^7^ To improve the management of CAP and increase patient survival, especially in the most severe cases, it is essential to understand the clinical picture, outcomes and potentially modifiable risk factors associated with mortality^8^

The term health care-associated pneumonia is no longer used because patients in health care facilities, nursing homes, dialysis centers, outpatient clinics, or patients with a history of hospitalization within the past 90-days should be treated for CAP unless specific risk factors that should prompt the use of broad-spectrum antimicrobial agents are present^9^ HAP is defined as an infection of the pulmonary tissue confirmed by imaging as a new pulmonary infiltrate that develops more than 48-hours after admission in non-intubated patients. Ventilator-Associated Pneumonia (VAP) is defined as pneumonia that develops after 48-hours of endotracheal intubation. Diagnosing HAP and VAP also requires the presence of productive cough, fever, leukocytosis, purulent tracheobronchial secretions, and a reduction in the partial pressure of oxygen (PaO_2_)/FiO_2_. Nosocomial pathogens colonizing the oropharynx and the aspiration of oropharyngeal contents significantly contribute to the occurrence of HAP and VAP^9^

The risk of acquiring HAP and VAP increases significantly in patients under mechanical ventilation and can be associated with high mortality rates and greater morbidity due to delays in diagnosis and adequate treatment^5^^,^^10^, ^11^, ^12^ The frequency of pathogens may vary among institutions, but the most frequent bacterial agents of HAP are *Pseudomonas aeruginosa, Acinetobacter* spp., *Klebsiella pneumoniae, Escherichia coli, Enterobacter* spp.) and *Staphylococcus aureus*^13^

In addition to imaging tests, which are essential in the diagnosis of pneumonia, and biochemical markers that allow the calculation of severity scores, rapid multiplex molecular panels tests have recently been introduced into clinical practice. These tests allow rapid etiological diagnosis and support the rational use of antimicrobial agents. On the other hand, these methods are expensive and should therefore be used based on guidelines and risk factors. Additionally, early identification of the agent causing pneumonia significantly contributes to the rational use of antimicrobial agents, potentially reducing complications and mortality.

In this sense, the main objective of this review is to propose an algorithm for the rational use of laboratory tests for the diagnosis of severe CAP, hospital-acquired pneumonia, and VAP.

## Materials and methods

**Fig. 1:**
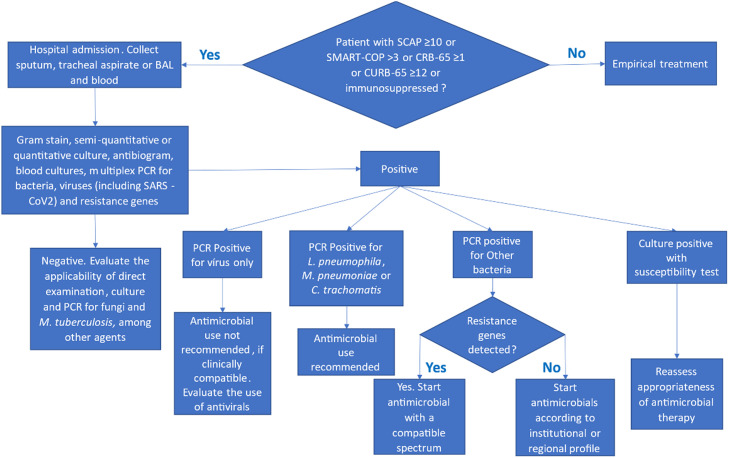
Diagnostic algorithm for community-acquired pneumonia (CAP) and Healthcare-Associated Pneumonia (HAP). This flowchart outlines the steps for the microbiological diagnosis of CAP and HAP, considering disease severity and individual risk factors. CAP, Community-Acquired Pneumonia; HAP, Healthcare-Associated Pneumonia.

**Fig. 2:**
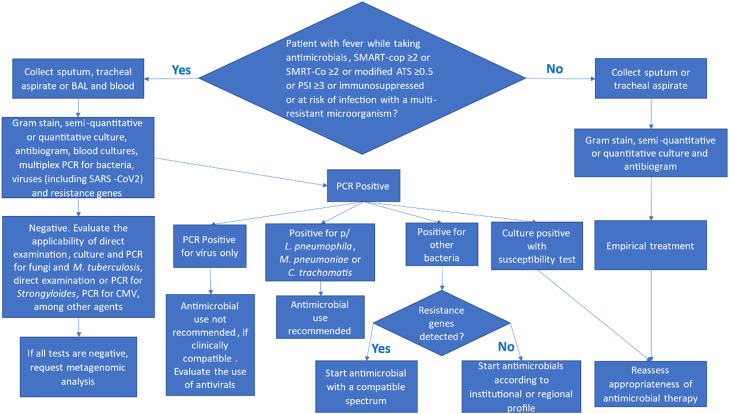
Diagnostic algorithm for ventilator-associated pneumonia (VAP). This flowchart presents the recommended approach for microbiological investigation of VAP, emphasizing the early identification of the etiological agent to optimize antimicrobial therapy. VAP, Ventilator-Associated Pneumonia.

This review analyzed comparative studies that evaluated the clinical and laboratory diagnosis of severe CAP, HAP, and pneumonia associated with mechanical ventilation. The bibliographic review was guided by two questions and by flowcharts constructed by experts (Figs 1 and 2). The inclusion criteria for studies focused on those evaluating microbiological diagnostic methods for severe pneumonia in adults, specifically Community-Acquired Pneumonia (CAP), Hospital-Acquired Pneumonia (HAP), and Ventilator-Associated Pneumonia (VAP). Studies were selected based on their relevance to the diagnostic algorithms proposed by the expert panel. Data synthesis involved a qualitative assessment of the evidence, focusing on the utility and limitations of various diagnostic approaches in different clinical scenarios. This approach allowed for a comprehensive overview of current practices and emerging technologies.

1. Which microbiological tests should be ordered if severe CAP, HAP, and pneumonia associated with mechanical ventilation are suspected?
2. How important is differential diagnosis for the appropriate clinical management of severe CAP, HAP, and pneumonia associated with mechanical ventilation?

## Results

Which microbiological tests should clinicians order if they suspect severe CAP, HAP, or ventilator-associated pneumonia (VAP)?

The diagnostic tests requested in cases of suspected community pneumonia are directly dependent on the severity score or the duration of immunosuppression. In HAP and pneumonia associated with mechanical ventilation, the tests to be requested will depend on the severity score, the duration of immunosuppression, and the risk of infection by multiresistant microorganisms. It is important to emphasize that the decision about which tests to order must be based on the patient's clinical assessment and the guidelines presented in the flowchart above.

### Gram stain

Microscopy using the Gram staining is essential for evaluating the quality of tracheal secretions and sputum samples. A representative lower respiratory tract sample should have fewer than 10 squamous epithelial cells and 25 or more polymorphonuclear leukocytes per microscopic field at 100 × magnification. When released quickly and performed by a trained professional, the absence of grouped Gram-positive cocci has a high negative predictive value (82 % to 100 %) for *Staphylococcus aureus* pneumonia^14^^,^^15^ Gram-negative bacilli are more difficult to visualize because they can be confused with debris. Therefore, it is crucial to combine Gram test results with other diagnostic methods^16^^,^^17^ In summary, the major concerns of Gram staining of sputum are its low reliability and subjective interpretability. The limited value of the sputum Gram stain test is due to the difficulty of obtaining samples or, more precisely, good-quality samples^18^

### Quantitative or semiquantitative culture

The great advantage of culturing material from the respiratory tract is that once it is positive, it allows antimicrobial susceptibility testing. Another advantage is the detection of bacterial species not included as targets in molecular panels^19^^,^^20^ Quantitative culture provides information on colony counts and can help differentiate between colonization and infection^21^^,^^22^ The greatest limitation of culture is the time required to release definitive results, which is at least 48 hours and, in some cases, up to 96 hours^23^

### Blood cultures

Clinicians should collect blood cultures from patients with severe CAP or inpatients with fever who are taking antimicrobials, have SMART-cop ≥2 or SMRT-Co ≥2 or modified ATS ≥0.5 or PSI ≥3, are immunosuppressed, or are at risk of infection with a multiresistant microorganism. With recent advances in reducing the contamination rate to near zero using Steripath® or similar devices, the specificity of blood cultures has increased^24^ Although blood culture has low sensitivity for HAP or VAP (15 % for VAP), approximately 25 % of bacteremic patients with VAP have positive blood cultures showing a nonpulmonary source of infection, and may be very useful for managing the patient^13^^,^^16^

The volume of blood collected and whether clinicians perform the collection before starting antimicrobial therapy greatly impact the test's sensitivity. In adults, it is recommended to collect 40 mL of blood, 20 mL per puncture, and 10 mL per blood culture bottle. At least two aerobic vials should be collected. The use of automated systems increases the sensitivity of blood cultures and speeds up the processing of positive cultures. Once the automated system detects a positive result, the laboratory should promptly perform microscopy on the culture broth and report the result to the attending physician within 1-hour^25^

### Antimicrobial susceptibility testing

Antimicrobial susceptibility testing allows the adequacy of empirical treatment to be reassessed, reducing or expanding the spectrum of antimicrobial therapy. Additionally, it can confirm whether the identified bacterial agents harbor the detected resistance genes, since molecular tests cannot determine whether the main pathogen or a colonizer carries the resistance gene^19^^,^^26^ As molecular panels present a limited number of detected genes, antimicrobial susceptibility tests complement them, as they allow the detection of relevant mechanisms not detected by the panels, such as resistance to polymyxins due to mutations in the *mgrB* gene and antimicrobial resistance due to mutations in porins.

The greatest limitation of the test is the total time required for its release, which includes the time for primary isolation plus 16 to 20 hours for growth to occur to read the antibiogram, except in blood cultures, for which it is possible to perform and read the test in a shorter period of time^27^

### Molecular panels

There are two types of molecular panels available in Brazil: those that require the classic structure of a molecular biology laboratory and are not capable of being carried out in an emergency laboratory, and those that consist of closed automated systems for detecting the most frequent pathogens of an infectious entity. In addition to the simultaneous detection of multiple pathogens, these closed systems allow the test to be available 24 hours a day, the test time is 70 minutes, they do not require the classic structure of a molecular biology laboratory divided into three areas, and they do not require an analyst specializing in molecular methods. The commercial closed system panel for diagnosing pneumonia currently available in Brazil is BioFire® FilmArray® Pneumonia (bioMérieux). This panel, in addition to identifying bacterial agents of atypical pneumonias – *Legionella pneumophila, Mycoplasmoides pneumoniae* and *Chlamydia pneumoniae* – identifies and provides an estimate of the infectious load for *S. pneumoniae, S. aureus, H. influenzae, Enterobacterales* and nonfermenting Gram-negative bacilli^28^ Results from this panel assist physicians in the decision to consider significant when two or more microorganisms are detected simultaneously. This panel showed a sensitivity of 90 % and 92 % and a specificity of 94 % and 97 % for health care-associated pneumonia and CAP, respectively^29^, ^30^, ^31^

The implementation of this panel has a relevant impact on antimicrobial therapy, leading to changes in empirical prescription in approximately 37 % of cases^30^

The other options for rapid multiplex molecular panels of respiratory tract infections available on the Brazilian market, the Allplex™ Respiratory Panel 4 (SeeGene) and the Xgen Sepse Kit (Mobius), require the structure of a traditional molecular biology laboratory and are not fully automated. Consequently, they are not considered rapid molecular tests and were not included in this manuscript.

### RT-PCR for SARS-CoV-2

RT-PCR, which converts RNA to cDNA and amplifies DNA through polymerase chain reaction, is a widely used method for detecting viral RNA in clinical samples. Since SARS-CoV-2 is an RNA virus, this method has been extensively used in the diagnosis of COVID-19. A systematic review with meta-analysis showed that the performance of the PCR test for detecting the virus is comparable, regardless of the sample collection method, such as nasopharyngeal swabs, oropharyngeal swabs, saliva, and bronchoalveolar lavage^32^ However, the sensitivity of the PCR test may vary according to the time of collection in relation to the onset of symptoms, being greater in the first days after the onset of symptoms^32^^,^^33^ Several studies have shown the high sensitivity of RT-PCR (80 % to 95 %) and a specificity greater than 95 %^34^^,^^35^

### Metagenomics

Metagenomics (mNGS) has a high cost, and some protocols require at least 72 hours to release results; however, while conventional diagnostic methods have a low average rate of pathogen detection (45.8 %), mNGS has a higher rate (80.5 %). Recently, Charalampous et al.^36^ (2024) reported a median turnaround time of 6.7 hours to obtain mNGS reports using saponin-base host depletion and Nanopore® sequencing. They reported a sensitivity of 93 % and specificity of 81 % for the detection of clinically relevant pathogens compared with routine testing^36^ In a recent review, the pooled 90-day mortality in patients managed with mNGS was 22.4 %, while this value was 43.4 % for patients managed with conventional diagnostic methods. Additionally, treatment based on mNGS results significantly reduced the length of hospital stay and length of stay in the intensive care unit^37^ Although mNGS performed better than other tests, the abundance of pathogens and host cell content in each sample could affect its sensitivity. Despite the greater identification of pathogens by mNSG than by other conventional methods combined, potential false-negative results were observed due to the high abundance of host cells in these samples^38^

1 How important is differential diagnosis for the appropriate clinical management of severe CAP, HAP, and pneumonia associated with mechanical ventilation?

Bacteria, viruses, and fungi can cause pneumonia. In patients taking high-dose corticosteroids, *Strongyloides stercoralis* larvae may migrate to the lungs and cause infiltrates resembling pneumonia. For previously healthy patients with mild pneumonia, clinicians may use empirical treatment. However, for immunocompromised patients and those with comorbidities or risk of multidrug-resistant infections, identifying the causative agent becomes crucial for effective targeted therapy^16^ In this scenario, molecular tests can play a crucial role, allowing rapid and accurate identification of the etiological agent, in addition to detecting resistance genes. In addition to allowing rapid reassessment of antimicrobial therapy, the detection of resistance genes allows the implementation of contact precaution measures^39^, ^40^, ^41^ Clinical management based on molecular panels can reduce the length of hospital stay^41^^,^^42^ Despite their great potential, few studies have shown their clinical impact; most of them are retrospective, there is still no consensus regarding interpretation, and many studies have scarce data on pneumonia case definitions^43^

## Discussion

Diagnostic tests play a fundamental role in determining the appropriate treatment for severe pneumonia. Gram staining has a high negative predictive value for *S. aureus* pneumonia, but requires an experienced microbiology professional 24 hours a day. Bacterial culture is valuable for accurately identifying the causative agent but requires a minimum of 48 hours to obtain results. On the other hand, it allows the performance of antimicrobial susceptibility tests, which are essential for reassessing the adequacy of antimicrobial therapy. Despite their low sensitivity, blood cultures also allow for the diagnosis of infections originating from extrapulmonary sites, which may result in better patient care.

Rapid molecular panels, when used in cases of severe pneumonia, potentially allow optimization of empirical antimicrobial treatment, as well as provide an estimate of the bacterial load, which assists in interpreting the relevance of detected pathogens. Although rapid molecular panels were introduced relatively recently in clinical practice, a few studies have shown the clinical impact of PCR-based molecular panels for the diagnosis of pneumonia. Most studies are retrospective; there is still no consensus regarding the interpretation of results, and many studies have scarce data on pneumonia case definitions^39^^,^^40^^,^^44^ Despite these limitations, rapid molecular panels are essential tools for the accurate detection of respiratory pathogens in a short period, improving clinical management. The use of molecular panels for the diagnosis of pneumonia has been associated with a reduction in the length of hospital stay, a reduction in the length of stay in ICUs, and a reduction in the consumption of antimicrobial agents^41^

Despite their diagnostic potential, the cost is the main limitation. Particularly in resource-limited settings, where their high cost can be a barrier to broad implementation. Furthermore, although these tools offer high sensitivity and specificity, there is no universal consensus on how to interpret co-detections and semi-quantitative results, which may lead to either overtreatment or underestimation of relevant pathogens. Robust cost-effectiveness studies are still scarce, particularly in low- and middle-income countries, highlighting the need for context-specific evaluations.

The rational use of molecular panels for diagnosing severe pneumonia can significantly reduce hospital and ICU stays and 90-day mortality. These technologies offer rapid and accurate pathogen identification, enabling timely and targeted antimicrobial therapy. However, their optimal integration into clinical practice requires careful consideration of cost-effectiveness, particularly in resource-limited settings, and the establishment of clear interpretive guidelines. Future research should focus on conducting robust cost-effectiveness analyses of molecular panels in diverse healthcare environments and developing standardized protocols for their interpretation and clinical application. Further studies are also needed to explore the long-term impact of these diagnostic tools on patient outcomes and antimicrobial stewardship programs. Additionally, research into novel, more affordable diagnostic technologies and their accessibility in low-resource settings will be crucial for improving global pneumonia management.

## Limitations

While molecular panels offer significant advantages in terms of speed and breadth of pathogen detection, it is crucial to acknowledge their limitations and the ongoing debates surrounding their optimal use. A key challenge lies in the interpretation of results, particularly the clinical significance of detecting multiple pathogens or commensal organisms. There is a lack of clear consensus on the thresholds for interpreting pathogen load and differentiating between colonization, co-infection, and true infection, which can lead to diagnostic ambiguity and potentially inappropriate treatment decisions. This ambiguity is further compounded by the fact that molecular tests detect nucleic acids, not necessarily viable organisms, meaning a positive result does not always equate to an active infection requiring antimicrobial therapy.

Furthermore, the cost-effectiveness of implementing these advanced molecular panels, especially in resource-limited settings, remains a significant concern. The high initial investment in equipment and reagents, coupled with the need for specialized training and infrastructure, can be prohibitive for many healthcare systems. While studies have shown potential benefits in reducing hospital stays and optimizing antimicrobial use, the economic impact needs to be carefully weighed against the local epidemiological context and available resources. In environments with limited resources, a pragmatic approach that prioritizes essential diagnostic tools and considers the incremental benefit of molecular panels is often necessary. Future research should focus on developing clear interpretive guidelines and conducting robust cost-effectiveness analyses in diverse healthcare settings to ensure that the promise of molecular diagnostics is realized equitably and sustainably.

## Authors’ contributions

JLMS conceptualize the work and wrote the original draft. AC, DWCLS and APJ reviewed and edited the manuscript. APJ managed the resources for the study. All authors read and approved the final version of the manuscript.

## Declaration of competing interest

The authors declare no conflicts of interest.
